# The hepatocellular carcinoma modified Gustave Roussy Immune score (HCC‐GRIm score) as a novel prognostic score for patients treated with atezolizumab and bevacizumab: A multicenter retrospective analysis

**DOI:** 10.1002/cam4.5294

**Published:** 2022-09-26

**Authors:** Takeshi Hatanaka, Atsushi Naganuma, Atsushi Hiraoka, Toshifumi Tada, Masashi Hirooka, Kazuya Kariyama, Joji Tani, Masanori Atsukawa, Koichi Takaguchi, Ei Itobayashi, Shinya Fukunishi, Kunihiko Tsuji, Toru Ishikawa, Kazuto Tajiri, Hironori Ochi, Satoshi Yasuda, Hidenori Toyoda, Chikara Ogawa, Takashi Nishimura, Noritomo Shimada, Kazuhito Kawata, Hisashi Kosaka, Satoru Kakizaki, Takaaki Tanaka, Hideko Ohama, Kazuhiro Nouso, Asahiro Morishita, Akemi Tsutsui, Takuya Nagano, Norio Itokawa, Tomomi Okubo, Taeang Arai, Michitaka Imai, Yohei Koizumi, Shinichiro Nakamura, Masaki Kaibori, Hiroko Iijima, Yoichi Hiasa, Takashi Kumada, the Real‐life Practice Experts for HCC (RELPEC) Study Group, and HCC 48 Group (hepatocellular carcinoma experts from 48 clinics in Japan)

**Affiliations:** ^1^ Department of Gastroenterology Gunma Saiseikai Maebashi Hospital Maebashi Gunma Japan; ^2^ Department of Gastroenterology National Hospital Organization Takasaki General Medical Center Takasaki Japan; ^3^ Gastroenterology Center Ehime Prefectural Central Hospital Matsuyama Japan; ^4^ Department of Internal Medicine Japanese Red Cross Himeji Hospital Himeji Japan; ^5^ Department of Gastroenterology and Metabology Ehime University Graduate School of Medicine Ehime Japan; ^6^ Department of Gastroenterology Okayama City Hospital Okayama Japan; ^7^ Department of Gastroenterology and Hepatology Kagawa University Kagawa Japan; ^8^ Division of Gastroenterology and Hepatology, Department of Internal Medicine Nippon Medical School Tokyo Japan; ^9^ Department of Hepatology Kagawa Prefectural Central Hospital Takamatsu Japan; ^10^ Department of Gastroenterology Asahi General Hospital Asahi Japan; ^11^ Premier Departmental Research of Medicine Osaka Medical and Pharmaceutical University Osaka Japan; ^12^ Center of Gastroenterology Teine Keijinkai Hospital Sapporo Japan; ^13^ Department of Gastroenterology Saiseikai Niigata Hospital Niigata Japan; ^14^ Department of Gastroenterology Toyama University Hospital Toyama Japan; ^15^ Center for Liver‐Biliary‐Pancreatic Disease Matsuyama Red Cross Hospital Matsuyama Japan; ^16^ Department of Gastroenterology and Hepatology Ogaki Municipal Hospital Ogaki Japan; ^17^ Department of Gastroenterology Japanese Red Cross Takamatsu Hospital Takamatsu Japan; ^18^ Division of Gastroenterology and Hepatology, Department of Internal Medicine Hyogo Medical University Nishinomiya Japan; ^19^ Division of Gastroenterology and Hepatology Otakanomori Hospital Kashiwa Japan; ^20^ Hepatology Division, Department of Internal Medicine II Hamamatsu University School of Medicine Hamamatsu Japan; ^21^ Department of Surgery Kansai Medical University Hirakata Japan; ^22^ Department of Clinical Research National Hospital Organization Takasaki General Medical Center Takasaki Japan; ^23^ Department of Gastroenterology and Hepatology Gunma University Graduate School of Medicine Maebashi Japan; ^24^ Department of Nursing Gifu Kyoritsu University Ogaki Japan

**Keywords:** aspartate transaminase‐to‐alanine transaminase ratio, atezolizumab and bevacizumab, HCC‐GRIm score, hepatocellular carcinoma, lactate dehydrogenase, neutrophil‐to‐lymphocyte ratio

## Abstract

**Aim:**

This study investigated whether or not the hepatocellular carcinoma modified Gustave Roussy Immune Score (HCC‐GRIm‐Score) serves as a prognostic indicator for HCC patients treated with atezolizumab and bevacizumab (Atez/Bev).

**Methods:**

A total of 405 HCC patients who received Atez/Bev from September 2020 to January 2022 at 22 different institutions were included in this retrospective study. The HCC‐GRIm score was based on the combination of the albumin level (<3.5 g/L = 1 point), lactate dehydrogenase (≥245 U/L = 1 point), neutrophil‐to‐lymphocyte ratio (≥4.8 = 1 point), aspartate aminotransferase‐to‐alanine aminotransferase ratio (≥1.44 = 1 point), and total bilirubin level (≥1.3 mg/dl = 1 point). Patients were divided into the low‐score group (0, 1, or 2 points) and the high‐score group (3, 4, or 5 points).

**Results:**

There were 89 (22.0%), 141 (34.8%), 106 (26.2%), 49 (12.1%), 16 (4.0%), and 4 (1.0%) patients with scores of 0, 1, 2, 3, 4, 5, respectively. The progression‐free survival (PFS) in the low‐score group was significantly longer than that in the high‐score group (median 7.8 vs. 3.5 months, *p* < 0.001). The median overall survival (OS) of the low‐score group was not reached at the time cutoff, with a 1‐year survival rate of 75.5%, whereas the median OS of the high‐score group was 8.5 months, showing a significant difference (*p* < 0.001). A high HCC‐GRIm score was a significant unfavorable factor associated with the PFS and OS in multivariate analyses (*p* = 0.002 and *p* < 0.001, respectively).

**Conclusions:**

The HCC‐GRIm score serves as a novel prognostic score for HCC patients treated with Atez/Bev.

## Graphical abstract

The hepatocellular carcinoma modified Gustave Roussy Immune score (HCC‐GRIm‐Score), which was based on the combination of the albumin level, lactate dehydrogenase, neutrophil‐to‐lymphocyte ratio, aspartate aminotransferase‐to‐alanine aminotransferase ratio, and total bilirubin level, serves as a novel prognostic score for HCC patients treated with atezolizumab and bevacizumab.

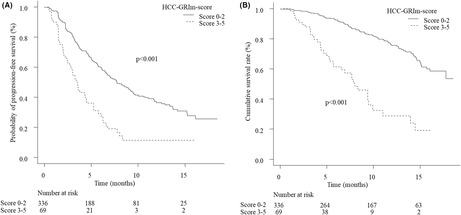

## 1 INTRODUCTION

Liver cancer is the seventh‐most frequently diagnosed malignant tumor and ranks second as cancer‐related death worldwide.^1^ Hepatocellular carcinoma (HCC) is the most frequent type of liver cancer.^2^ According to the latest Barcelona Clinic Liver Cancer (BCLC) guidelines,^3^, ^4^ systemic therapy was recommended as the first‐line treatment option for BCLC intermediate or advanced‐stage HCC. Imbrave150^5^ demonstrated that treatment with atezolizumab and bevacizumab (Atez/Bev), which is an antiprogrammed death ligand 1 inhibitor and a humanized antivascular endothelial growth factor monoclonal antibody (VEGF), resulted in a better progression‐free survival (PFS) and overall survival (OS) than sorafenib. Atez/Bev is thus recommended as the first‐line systemic therapy according to recent guidelines.^3^, ^4^

Bigot et al.^6^ reported that the Gustave Roussy Immune score (GRIm score) was developed and validated in patients with various types of malignant tumors receiving immune checkpoint inhibitors (ICIs). This scoring system consisted of the albumin level, lactate dehydrogenase (LDH) level, and neutrophil‐to‐lymphocyte ratio (NLR), which reflect the host immune system status. Patients with a low GRIm score (0 or 1) showed significantly better survival than those with a high score (>1).^6^ Accordingly, the GRIm score is expected to be useful for identifying patients likely to benefit from ICI treatment.^6^

Previous studies reported that the GRIm score was a promising biomarker for patients with non‐small‐cell lung cancer (NSCLC) receiving cytotoxic chemotherapy,^7^ epidermal growth factor receptor‐tyrosine kinase inhibitors,^7^ or immunotherapy.^8^ In addition, the preoperative GRIm score is also a prognostic indicator in patients with NSCLC,^9^ colorectal cancer,^10^ and esophageal squamous cell carcinoma^11^ after curative surgical treatment. Recently, Li et al. proposed the modified GRIm score (HCC‐GRIm score) and validated its prognostic ability in HCC patients treated with ICI monotherapy.^12^ The HCC‐GRIm score, which is based on the albumin level, LDH level, NLR, aspartate transaminase‐to‐alanine transaminase ratio (AST‐to‐ALT ratio), and total bilirubin level, showed a better prognostic performance than the original GRIm score.^12^ However, none of the patients included in that previous study^12^ received Atez/Bev treatment. Accordingly, the utility of the HCC‐GRIm score in patients receiving Atez/Bev remains uncertain.

The present study investigated whether or not the HCC‐GRIm score estimates the prognosis and provides a practical guide for Atez/Bev in HCC patients.

## 2 METHODS

### 2.1 Patients

**FIGURE 1:**
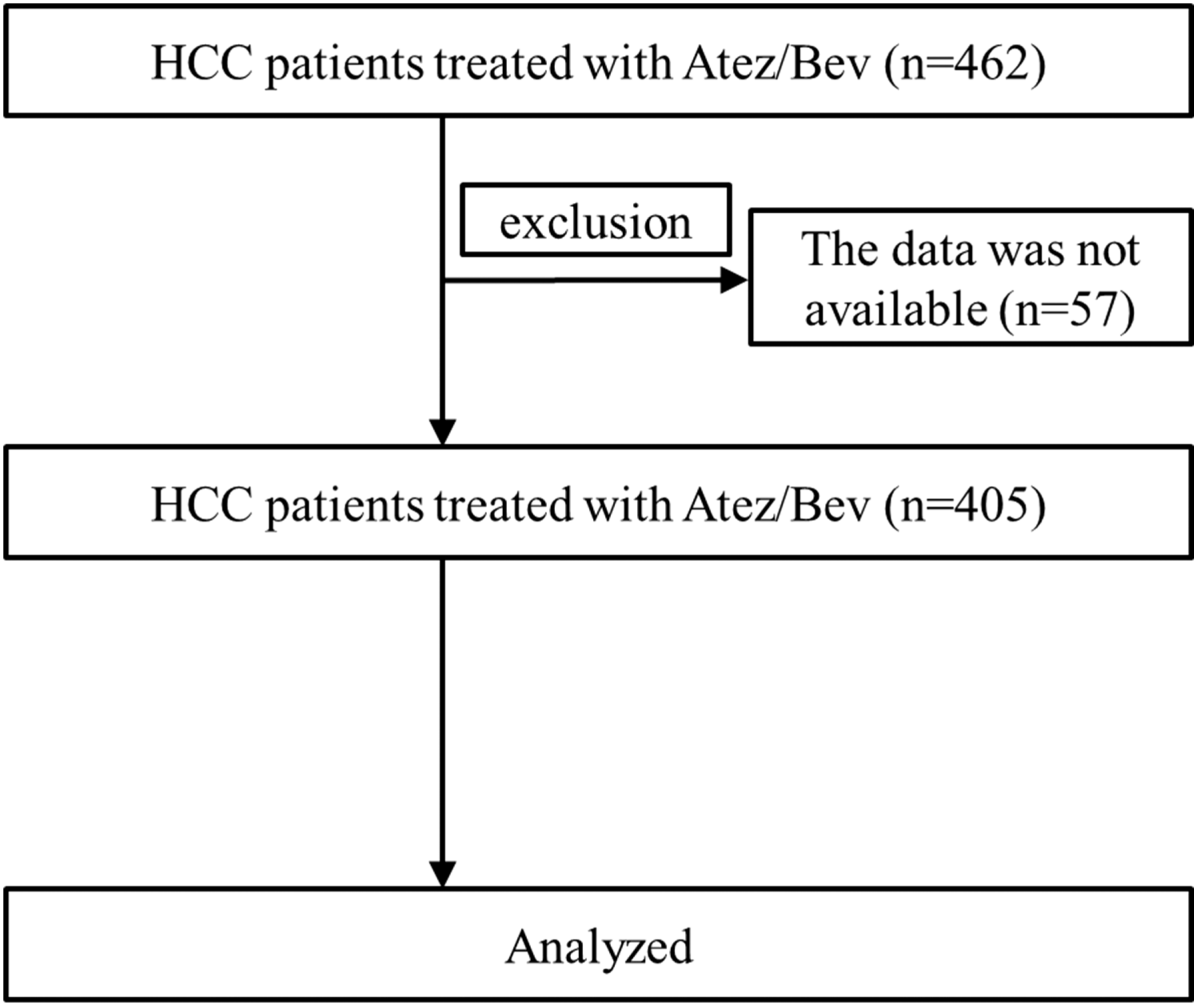
The eligible patient selection process.

A total of 462 HCC patients who received Atez/Bev from September 2020 to January 2022 at 22 different institutions were included in this study. Of these patients, we excluded 57 because their pretreatment laboratory data, including the AST (*n* = 2), LDH (*n* = 42), and NLR (*n* = 13), were not available. Accordingly, the remaining 405 patients were included in this retrospective cohort study (Figure 1).

All patients had a histological or radiological diagnosis of HCC based on the histological examination or typical radiological findings. We reviewed medical records, including laboratory data and radiological findings, and analyzed the clinical course. The BCLC staging system^4^ was used to evaluate the extent of disease progression. The preserved liver function and the severity of cirrhosis were assessed by the Child‐Pugh classification, albumin‐bilirubin (ALBI) score,^13^ and modified albumin‐bilirubin (mALBI) grade.^14^

### 2.2 Chronic liver disease definition

If patients were seropositive for antihepatitis C virus antibody (anti‐HCV ab) or hepatitis B surface antigen (HBs‐Ag), the cause of HCC was attributed to hepatitis B virus (HBV) or hepatitis C virus (HCV), respectively. If patients had significant alcohol consumption (≥60 g/day) and were negative for both anti‐HCV ab and HBs‐Ag, alcoholic liver injury was considered to have caused carcinogenesis. Nonalcoholic fatty liver disease (NAFLD) was diagnosed based on the histological evaluation of the liver specimen.^15^ When a liver specimen was not available, the etiology of patients with findings of fatty liver disease and with comorbidity of metabolic syndrome, including obesity, hypertension, and diabetes mellitus, were considered to be NAFLD. Viral‐related HCC was defined in patients who were positive for either anti‐HCV ab or HBs‐Ag, and nonviral‐related HCC was defined in patients negative for both of them.

### 2.3 Atez/Bev treatment

Patients were intravenously given 1200 mg atezolizumab and 15 mg/kg bevacizumab every 3 weeks until disease progression or unacceptable adverse events (AEs). Treatment‐related AEs were graded by The Common Terminology Criteria for Adverse Events version 5.0. When AEs developed, we assessed the causal attribution to each drug based on the published toxic profile. The radiological imaging assessment was performed by the Response Evaluation Criteria In Solid Tumors version 1.1 (RECIST ver.1.1). We defined the period from the initiation of Atez/Bev to disease progression or death as the PFS and that from the initiation of Atez/Bev to death as the OS.

### 2.4 Calculation of the HCC‐GRIm score

The HCC‐GRIm score was estimated as described in a previous study^12^; it was determined by the combination of the albumin level (<3.5 g/L = 1 point), LDH level (≥245 U/L = 1 point), NLR (≥4.8 = 1 point), AST‐to‐ALT ratio (≥1.44 = 1 point), and total bilirubin level (≥1.3 mg/dl = 1 point). The NLR was calculated by dividing the baseline peripheral neutrophil count by the lymphocyte count. The AST‐to‐ALT ratio was also calculated by dividing the serum level of AST by the serum level of ALT.

Based on their HCC‐GRIm scores, patients were divided into the low‐score group (0, 1, or 2 points) and the high‐score group (3, 4, or 5 points).

### 2.5 Statistical analyses

All statistical analyses were conducted using the EZR software program, Ver. 1.55 (Saitama Medical Center, Jichi Medical University, Saitama, Japan).^16^ The numerical and categorical variables were presented as the median with interquartile range and number with the percentage in parentheses, respectively. We used the Mann–Whitney *U* test, chi‐squared, or Fisher's exact test to conduct the statistical analyses. The PFS and OS curves were calculated by the Kaplan–Meier method and compared using the log‐rank test. We used the Cox proportional regression model to investigate the predictive factors associated with the PFS and OS. The following factors were included in multivariate analyses: age, male, etiology (nonviral), BCLC stage (C or D), mALBI grade (2b or 3), treatment line (later line), HCC‐GRIm score (≥3 points), α‐fetoprotein (AFP; ≥100 ng/ml), and des‐gamma‐carboxy prothrombin (DCP; ≥100 mAU/ml). The cutoff value of AFP was compatible with previous studies associated with immune classification,^17^ gene mutation,^18^ and clinical ICI treatment.^19^ We also estimated the hazard ratio (HR), 95% confidence interval (CI), and *p* value of each factor involved in the multivariate analyses. *p* values lower than 0.05 were considered statistically significant.

## 3 RESULTS

### 3.1 Patient characteristics according to the HCC‐GRIm score

Patient characteristics are shown in Table 1. The median age of the included patients was 74.0 (68.0–79.0) years old, and there were 328 (81.0%) males. Chronic liver diseases were HCV, HBV, alcohol, NAFLD, and others in 143 (35.3%), 65 (16.0%), 72 (17.8%), 75 (18.5%), and 50 (12.3%) patients, respectively. One hundred seven (75.9%) patients achieved eradication of HCV and nucleoside analog suppressed HBV in 47 (74.6%) patients at the time of initiation of Atez/Bev. The BCLC stage was determined to be very early, early, intermediate, advanced, and terminal in 5 (1.2%), 15 (3.7%), 148 (36.5%), 234 (57.8%), and 3 (0.7%) patients, respectively. The median ALBI score was −2.36 (−2.69 to −2.06), and the mALBI grade was 1, 2a, 2b, and 3 in 139 (34.3%), 99 (24.4%), 163 (40.2%), and 4 (1.0%), respectively. The majority of patients (92.3%) were Child‐Pugh class A. The median NLR was 2.59 (1.86–3.68). The values of AST, ALT, and LDH were 40 (28–5.7), 27 (19–40), and 210 (181–253) U/L, respectively.

**TABLE 1:** Patient characteristics

Factors	Overall patients (*n* = 405)	Low HCC‐GRIm score group (0–2 points, *n* = 336)	High HCC‐GRIm score group (3–5 points, *n* = 69)	*p* value
Age (years)	74.0 [68.0, 79.0]	74.0 [68.0, 79.0]	74.0 [68.0, 78.0]	0.8
Gender, *n* (%)				
Male	328 (81.0)	272 (81.0)	56 (81.2)	1
Chronic liver diseases, *n* (%)				
HCV	143 (35.3)	121 (36.0)	22 (31.9)	0.7
HBV	65 (16.0)	56 (16.7)	9 (13.0)	
Alcohol	72 (17.8)	56 (16.7)	16 (23.2)	
NAFLD	75 (18.5)	61 (18.2)	14 (20.3)	
Others	50 (12.3)	42 (12.5)	8 (11.6)	
BCLC stage, *n* (%)				
Very early	5 (1.2)	4 (1.2)	1 (1.4)	0.2
Early	15 (3.7)	14 (4.2)	1 (1.4)	
Intermediate	148 (36.5)	125 (37.2)	23 (33.3)	
Advanced	234 (57.8)	192 (57.1)	42 (60.9)	
Terminal	3 (0.7)	1 (0.3)	2 (2.9)	
SVR, *n* (%)	107 (75.9)	92 (77.3)	15 (68.2)	0.4
Nucleoside analog for HBV, *n* (%)	47 (74.6)	40 (72.7)	7 (87.5)	0.7
Total bilirubin (mg/dl)	0.80 [0.60, 1.00]	0.80 [0.60, 1.00]	0.80 [0.68, 1.50]	0.01
Albumin (g/dl)	3.7 [3.3, 4.1]	3.8 [3.5, 4.1]	3.2 [3.0, 3.4]	<0.001
ALBI score	−2.36 [−2.69, −2.06]	−2.51 [−2.74, −2.19]	−1.92 [−2.14, −1.72]	<0.001
mALBI grade, *n* (%)				
1	139 (34.3)	138 (41.1)	1 (1.4)	<0.001
2a	99 (24.4)	93 (27.7)	6 (8.7)	
2b	163 (40.2)	104 (31.0)	59 (85.5)	
3	4 (1.0)	1 (0.3)	3 (4.3)	
Child‐Pugh classification, *n* (%)				
A	374 (92.3)	319 (94.9)	55 (79.7)	<0.001
B	30 (7.4)	17 (5.1)	13 (18.8)	
C	1 (0.2)	0 (0.0)	1 (1.4)	
Treatment line, *n* (%)				
First line	248 (61.2)	208 (61.9)	40 (58.0)	0.6
Later line	157 (38.8)	128 (38.1)	29 (42.0)	
Macroscopic vascular invasion, *n* (%)	78 (19.3)	58 (17.3)	20 (29.0)	0.03
Extrahepatic spread, *n* (%)	133 (32.8)	114 (33.9)	19 (27.5)	0.33
Neutrophils (/μl)	2920 [2179, 3927]	2912 [2211, 3881]	2937 [2025, 4520]	0.7
Lymphocytes (/μl)	1130 [780, 1519]	1172 [888, 1557]	770 [474, 1188]	<0.001
NLR	2.59 [1.86, 3.68]	2.46 [1.79, 3.44]	3.67 [2.58, 5.19]	<0.001
PLR	123 [89, 186]	117 [84, 169]	212 [123, 344]	<0.001
Platelet (10^4^/μl)	13.8 [10.6, 18.5]	13.8 [10.5, 17.9]	15.8 [10.8, 23.3]	0.08
AST (U/L)	40 [28, 57]	37 [27, 53]	56 [40, 89]	<0.001
ALT (U/L)	27 [19, 40]	27 [19, 40]	28 [20, 38]	0.8
AST‐to‐ALT ratio	1.46 [1.16, 1.83]	1.35 [1.11, 1.67]	1.86 [1.65, 2.65]	<0.001
LDH (U/L)	210 [181, 253]	204 [178, 233]	276 [253, 335]	<0.001
AFP ≥100 ng/ml, *n* (%)	171 (42.2)	130 (38.7)	41 (59.4)	0.002
DCP ≥100 mAU/ml, *n* (%)	264 (65.3)	203 (60.4)	61 (89.7)	<0.001
Albumin <3.5 g/dl, *n* (%)	161 (39.8)	98 (29.2)	63 (91.3)	<0.001
LDH ≥245 U/L, *n* (%)	113 (27.9)	57 (17.0)	56 (81.2)	<0.001
NLR ≥4.8, *n* (%)	48 (11.9)	25 (7.4)	23 (33.3)	<0.001
AST‐to‐ALT ratio ≥1.44, *n* (%)	210 (51.9)	143 (42.6)	67 (97.1)	<0.001
Total bilirubin ≥1.3 mg/dl, *n* (%)	52 (12.8)	30 (8.9)	22 (31.9)	<0.001
HCC‐GRIm score, *n* (%)				
0	89 (22.0)	89 (26.5)	0 (0.0)	<0.001
1	141 (34.8)	141 (42.0)	0 (0.0)	
2	106 (26.2)	106 (31.5)	0 (0.0)	
3	49 (12.1)	0 (0.0)	49 (71.0)	
4	16 (4.0)	0 (0.0)	16 (23.2)	
5	4 (1.0)	0 (0.0)	4 (5.8)	

Abbreviations: AFP, α‐fetoprotein; AlBI score, albumin‐bilirubin score; ALT, alanine aminotransferase; AST, aspartate aminotransferase; BCLC stage, Barcelona Clinic Liver Cancer stage; DCP, des‐gamma‐carboxy prothrombin; HBV, hepatitis B virus; HCC‐GRIm score, hepatocellular carcinoma Gustave Roussy Immune score; HCV, hepatitis C virus; LDH, lactate dehydrogenase; mALBI grade, modified albumin‐bilirubin grade; NAFLD, non‐alcoholic fatty liver disease; NLR, neutrophil‐to‐lymphocyte ratio; PLR, platelet‐to‐lymphocyte ratio.

Regarding the HCC‐GRIm score, there were 89 (22.0%), 141 (34.8%), 106 (26.2%), 49 (12.1%), 16 (4.0%), and 4 (1.0%) patients with scores of 0, 1, 2, 3, 4, and 5, respectively. Accordingly, 336 (83.0%) patients were assigned to the low‐score group, and the remaining 69 (17.0%) were assigned to the high‐score group. The Child‐Pugh classification and mALBI grade were better in the low‐score group than in the high‐score group. The percentages of patients with macroscopic vascular invasion, AFP ≥100 ng/ml, and DCP ≥100 mAU/ml were significantly lower in the low‐score group than in the high‐score group.

### 3.2 Therapeutic efficacy according to the HCC‐GRIm score

Among patients in the low‐score group, the objective response assessed by RECIST ver.1.1 was complete response, partial response, stable diseases, progressive disease, and nonevaluable in 9 (2.7%), 78 (23.2%), 158 (47.0%), 54 (16.1%), and 37 (11.0%), respectively, which differed significantly from those in the high‐score group (*p* = 0.039). The objective response rate (ORR) and disease control rate (DCR) were numerically higher in the low‐score group than in the high‐score group (*p* = 0.2 and 0.08, respectively; Table 2).

**TABLE 2:** The objective response according to the HCC‐GRIm score

Factors	Low HCC‐GRIm score group (0–2 points, *n* = 336)	High HCC‐GRIm score group (3–5 points, *n* = 69)	*p* value
Objective response, *n* (%)			
CR	9 (2.7)	1 (1.4)	0.039
PR	78 (23.2)	11 (15.9)	
SD	158 (47.0)	31 (44.9)	
PD	54 (16.1)	22 (31.9)	
NE	37 (11.0)	4 (5.8)	
Objective response rate (%)	25.9	17.4	0.2
Disease control rate (%)	72.9	62.3	0.08

Abbreviations: CR, complete response; HCC‐GRIm score, hepatocellular carcinoma Gustave Roussy Immune score; NE, nonevaluable; PD, progressive disease; PR, partial response; SD, stable disease.

**FIGURE 2:**
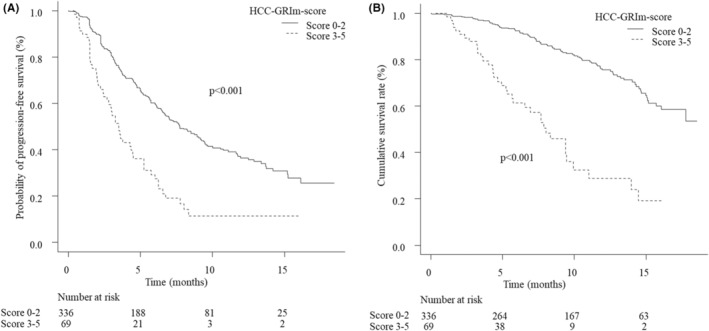
(A) Kaplan–Meier curve of the progression‐free survival (PFS). The median PFS was 7.8 (95% confidence interval [CI] 6.6–9.3) months in the low‐score group and 3.5 (95% CI 2.7–4.5) months in the high‐score group, with a significant difference (*p* < 0.001). (B) Kaplan–Meier curve of the overall survival (OS). The median OS of the low‐score group was not reached, with a 1‐year survival rate of 75.5% (95% CI 69.3–80.6), while the median OS was 8.0 (95% CI 8.5–9.9) months in the high‐score group. The low‐score group showed a significantly better OS than the high‐score group (*p* < 0.001).

**FIGURE 3:**
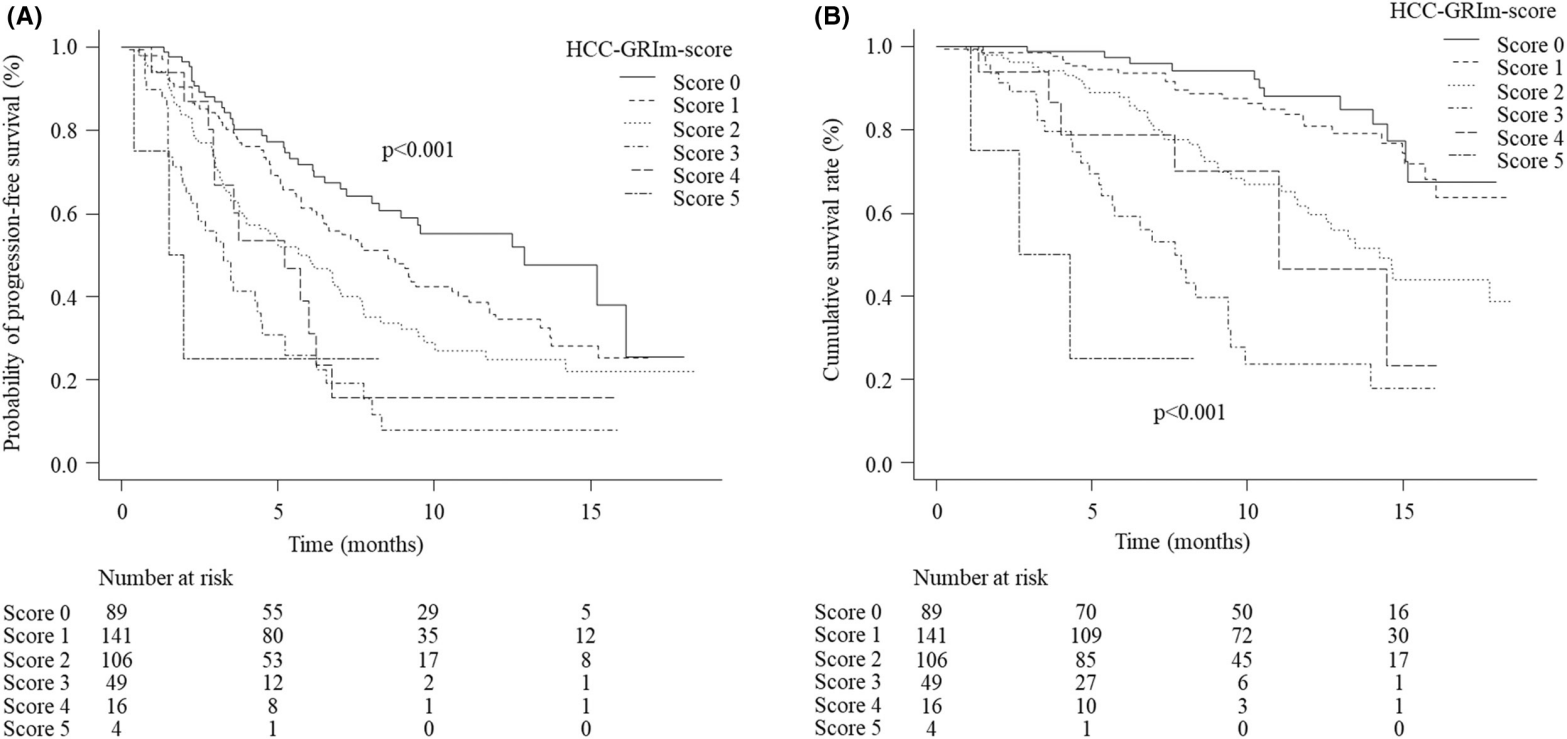
The progression‐free survival (A) and overall survival (B) according to each hepatocellular carcinoma Gustave Roussy Immune score.

At the time of the analysis, 237 PFS events (58.5%) were found, and 115 (28.4%) patients had died. The median PFS values of the low‐ and high‐score groups were 7.8 (95% CI 6.6–9.3) and 3.5 (95% CI 2.7–4.5) months, respectively. The PFS in the low‐score group was significantly longer than in the high‐score group (*p* < 0.001; Figure 2A). The median OS in the low‐score group was not reached at the time cutoff, with a 1‐year survival rate of 75.5% (95% CI 69.3–80.6); in contrast, the median OS of the high‐score group was 8.5 (95% CI 5.6–9.9) months. The OS in the low‐score group was significantly better than that in the high‐score group (*p* < 0.001; Figure 2B). Multivariate analyses showed that inclusion in the high‐score group was a significant unfavorable factor associated with the PFS and OS (PFS: HR 1.77, 95% CI 1.22–2.57, *p* = 0.003; OS: HR 2.44, 95% CI 1.54–3.89, *p* < 0.001; Table 3). According to the analysis of PFS based on each HCC‐GRIm score, the median PFS was 12.9 (95% CI 8.3 to NA) months, 8.5 (95% CI 6.4–10.8) months, 6.0 (95% CI 3.9–7.0) months, 3.3 (95% CI 2.1–4.5) months, 5.3 (95% CI 3.0–6.3) months, and 1.8 (95% CI 0.4 to NA) months in patients with the HCC‐GRIm score of 0, 1, 2, 3, 4, and 5 points, respectively, which amounted to statistically significant (*p* < 0.001; Figure 3A). The median OS was not reached in patients with the HCC‐GRIm score of 0 and 1 point, with a 1‐year survival rate of 88.1% (95% CI 76.4–94.3) and 80.8% (95% CI 71.2–87.5), and it was 14.2 (95% CI 11.6 to NA) months, 7.6 (95% CI 5.3–9.4) months, 11.0 (95% CI 4.0 to NA) months, and 3.5 (95% CI 1.1 to NA) months in patients with the HCC‐GRIm score of 2, 3, 4, and 5 points, respectively (*p* < 0.001; Figure 3B).

**TABLE 3:** Multivariate analyses associated with the PFS and OS

Factors		Analysis of the PFS		Analysis of the OS	
		HR (95% CI)	*p* value	HR (95% CI)	*p* value
Age	Per 1 year	1.01 (0.99–1.02)	0.4	1.02 (1.00–1.05)	0.03
Gender	Male	0.74 (0.52–1.04)	0.09	1.14 (0.72–1.82)	0.6
Etiology	Nonviral	1.04 (0.80–1.35)	0.8	0.90 (0.62–1.32)	0.6
BCLC stage	C or D stage	1.04 (0.79–1.36)	0.8	1.38 (0.92–2.09)	0.1
mALBI grade	2b or 3	1.36 (1.01–1.83)	0.04	2.10 (1.37–3.22)	<0.001
Treatment line	Later line	1.07 (0.82–1.39)	0.6	0.94 (0.64–1.38)	0.8
HCC‐GRIm score	≥3 points	1.77 (1.22–2.57)	0.003	2.44 (1.54–3.89)	<0.001
AFP	≥100 ng/ml	1.64 (1.24–2.16)	<0.001	1.54 (1.04–2.30)	0.03
DCP	≥100 mAU/ml	1.23 (0.90–1.69)	0.2	1.66 (0.99–2.76)	0.053

Abbreviations: AFP, α‐fetoprotein; BCLC stage, Barcelona Clinic Liver Cancer stage; CI, confidence interval; DCP, des‐gamma‐carboxy prothrombin; HCC‐GRIm score, hepatocellular carcinoma Gustave Roussy Immune score; HR, hazard ratio; mALBI grade, modified albumin‐bilirubin grade; OS, overall survival; PFS, progression‐free survival.

We reported the results of subgroup analyses of the PFS in patients with BCLC intermediate (Figure S1A), those with advanced stage (Figure S1B), those with BCLC intermediate or advanced stage and Child‐Pugh class A (Figure S1C), and those receiving first‐line treatment (Figure S1D). We also showed results of OS in those with BCLC intermediate (Figure S2A), those with advanced stage (Figure S2B), those with BCLC intermediate or advanced stage and Child‐Pugh class A (Figure S2C), and those receiving first‐line treatment (Figure S2D). Furthermore, we described the PFS and survival curve according to each HCC‐GRIm‐Score in Figure 3A,B.

### 3.3 Adverse events

The most common AE during observation was any‐grade proteinuria (*n* = 136, 33.6%), followed by any‐grade fatigue (*n* = 98, 24.2%), any‐grade appetite loss (*n* = 93, 23.0%), and any‐grade hypertension (*n* = 63, 15.6%: Table 4). Significant differences were not observed between the low‐ and high‐score groups, except for in any‐grade liver injury (*p* = 0.01).

**TABLE 4:** Adverse events according to the HCC‐GRIm‐score

Factors	Low HCC‐GRIm score group (0–2 points, *n* = 336)	High HCC‐GRIm score group (3–5 points, *n* = 69)	*p* value
Liver injury, *n* (%)			
Any grade	30 (8.9)	14 (20.3)	0.01
Grade ≥3	7 (2.1)	5 (7.2)	0.04
Hypertension, *n* (%)			
Any grade	53 (15.8)	10 (14.5)	0.9
Grade ≥3	14 (4.2)	2 (2.9)	1
Gastrointestinal hemorrhaging, *n* (%)			
Any grade	6 (1.8)	0 (0.0)	0.6
Grade ≥3	5 (1.5)	0 (0.0)	0.6
Appetite loss, *n* (%)			
Any grade	71 (21.1)	22 (31.9)	0.06
Grade ≥3	10 (3.0)	3 (4.3)	0.5
Proteinuria, *n* (%)			
Any grade	115 (34.2)	21 (30.4)	0.6
Grade ≥3	34 (10.1)	7 (10.1)	1.0
Fever, *n* (%)			
Any grade	21 (6.2)	8 (11.6)	0.1
Grade ≥3	6 (1.8)	0 (0.0)	0.6
Fatigue, *n* (%)			
Any grade	75 (22.3)	23 (33.3)	0.06
Grade ≥3	7 (2.1)	0 (0.0)	0.6
Edema or ascites, *n* (%)			
Any grade	37 (11.0)	6 (8.7)	0.7
Grade ≥3	13 (3.9)	1 (1.4)	0.5

Abbreviation: HCC‐GRIm score, hepatocellular carcinoma Gustave Roussy Immune score.

## 4 DISCUSSION

The GRIm score was first reported to improve the selection of patients likely to benefit from ICI treatment, as the efficacy of this treatment is limited.^6^ The GRIm score, based on the NLR, LDH level, and albumin level, is a useful tool for predicting the survival of patients with various types of malignant tumors (not including HCC).^6^ Therefore, to adapt the GRIm score for HCC, Li et al. proposed a modified scoring system, named the HCC‐GRIm score.^12^ They identified two liver‐specific factors—the AST‐to‐ALT ratio and total bilirubin level—as prognostic factors and integrated them into the HCC‐GRIm score.^12^ Those authors then demonstrated that the HCC‐GRIm score had a better predictive ability in HCC patients treated with various ICI regimens than the GRIm score.^12^ However, the HCC‐GRIm score was not evaluated in a cohort of HCC patients receiving Atez/Bev, which is a combination therapy of ICI and anti‐VEGF therapy. To our knowledge, this is the first study evaluating the prognostic value of the HCC‐GRIm score for patients receiving Atez/Bev, demonstrating that the HCC‐GRIm score serves as a prognostic factor of the PFS and OS.

The serum level of albumin reflects the nutritional status. Serum albumin and total bilirubin levels are also well‐known factors for evaluating preserved liver function. The Child‐Pugh classification included these two factors, and the ALBI score was calculated using them.^13^ The liver function largely contributed to the clinical outcome of systemic therapies, including atezolizumab plus bevacizumab,^20^ lenvatinib,^21^ sorafenib,^22^ and ramucirumab.^23^, ^24^ In addition, the liver function was also a predictive factor relevant to the transition of the postprogression treatment after sorafenib^25^ and lenvatinib.^26^ These previous reports indicated that a decreased albumin level and increased total bilirubin level were negative factors associated with the PFS and OS, which agreed with the present results.

Inflammation is a hallmark of cancer progression and a key component of the tumor microenvironment.^27^, ^28^ The NLR is a sensitive inflammatory biomarker. A recent study reported that the inflammatory gene signature and NLR were relevant to the clinical outcome in nivolumab‐treated HCC patients.^29^ Regarding Atez/Bev treatment, HCC patients with a low pretreatment NLR showed a better clinical outcome and survival than those with a high NLR.^30^, ^31^, ^32^ Another famous inflammatory biomarker is platelet‐to‐lymphocyte ratio (PLR). A previous study reported that patients with a low PLR showed a longer PFS than those with a high PLR.^32^In deed, our cohort showed that the PFS and OS in patients with a low PLR (<120) were better than those in patients with a high PLR (≥120; Figure S3A,B). Accordingly, inflammation plays an important role in the clinical outcome of Atez/Bev treatment.

Lactate dehydrogenase is commonly found in various tissues in the human body and is also a classic inflammatory marker. It is released by rapidly growing tumors, and a high value reflects a high tumor burden. A meta‐analysis investigated the relationship between the pretreatment LDH level and the clinical outcome in ICI‐treated NSCLC patients, demonstrating that patients with an elevated LDH value resulted in poor PFS and OS.^33^ Regarding HCC, a meta‐analysis revealed that the LDH level is a prognostic indicator for HCC patients receiving various treatments, such as surgical resection and transarterial chemoembolization.^34^ Although the role of LDH in ICI‐treated HCC patients remains uncertain, LDH may predict the survival in patients receiving Atez/Bev treatment.

A previous study reported that an elevated AST‐to‐ALT ratio was a negative prognostic indicator for HBV‐related HCC patients undergoing hepatectomy.^35^ Although the underlying mechanisms remain unclear, one speculated mechanism is that aggressive tumor progression results in a dramatic increase in the AST level and the clearance of the AST level decreases as the liver function becomes increasingly impaired.^35^ In brief, an elevated AST‐to‐ALT ratio may reflect both increased tumor progression activity and impaired liver function. Accordingly, the AST‐to‐ALT ratio may have prognostic value in HCC patients receiving Atez/Bev. In summary, the HCC‐GRIm score may reflect both the preserved liver function and cancer‐related inflammation.

With respect to PFS, poor liver function, the high value of NLR,^30^ and the elevated LDH^34^ are negative predictive factors associated with PFS, as we mentioned above. Because the HCC‐GRIm score comprised of these factors, the PFS in the low‐score group was significantly better than that in the high‐score group in the present study. However, the ORR and DCR did not show statistical significance. The lack of statistical significance might have been due to the low statistical power. Including more subjects might have affected the present results.

Recently, the utility of the C‐reactive protein (CRP) and alpha‐fetoprotein in immunotherapy score (CRAFITY score) is reported.^36^ This score consists of the baseline serum level of AFP and CRP and predicts the survival in HCC patients receiving various ICI treatments. We also reported that the CRAFITY score can predict the PFS as well as the OS in patients treated with Atez/Bev treatment.^19^ We compared the predictive performance of the HCC‐GRIm score and the CRAFITY score in the present cohort, indicating that the discrimination performance of the HCC‐GRIm score is almost equal to that of the CRAFITY score (Figure S4A,B). In addition, we also analyzed the predictive performance of the HCC‐GRIm score and ALBI grade, showing that the HCC‐GRIm score has significantly higher AUROC than the ALBI grade (Figure S5A,B). This is possible because the ALBI grade only represents the preserved liver function while the HCC‐GRIm score may reflect both the preserved liver function and cancer‐related inflammation, as we mentioned above. This difference may lead to the higher AUROC values of the HCC‐GRIm score than that of the ALBI grade.

With respect to AEs, we did not clearly explain why the incidence and severity of liver injury differed significantly between the low‐ and high‐score groups. Given that the HCC‐GRIm score included the LDH level and AST‐to‐ALT ratio, the high‐score group may have been more prone to developing liver damage than the low‐score group.

There were several limitations associated with the present study. First, despite the multicenter study, we did not avoid the selection bias due to a retrospective manner and relatively small sample size. Second, because the observation period in the present study was insufficient, a long observation period may have changed the present results. Third, about 7% of patients with the Child‐Pugh class B or C and about 40% of patients treated with Atez/Bev as later‐line treatment were included in the present study.

In conclusion, the HCC‐GRIm score serves as a novel prognostic score for HCC patients treated with Atez/Bev treatment.

## AUTHOR CONTRIBUTIONS

**Takeshi Hatanaka:** Conceptualization (lead); data curation (equal); formal analysis (lead); writing – original draft (lead). **Atsushi Naganuma:** Conceptualization (equal); data curation (equal); writing – review and editing (equal). **Atsushi Hiraoka:** Conceptualization (equal); data curation (equal); writing – review and editing (equal). **Toshifumi Tada:** Conceptualization (equal); data curation (equal); writing – review and editing (equal). **Masashi Hirooka:** Data curation (equal). **Kazuya Kariyama:** Data curation (equal). **Joji Tani:** Data curation (equal). **Masanori Atsukawa:** Data curation (equal). **Koichi Takaguchi:** Data curation (equal). **Ei Itobayashi:** Data curation (equal). **Shinya Fukunishi:** Data curation (equal). **Kunihiko Tsuji:** Data curation (equal). **Toru Ishikawa:** Data curation (equal). **Kazuto Tajiri:** Data curation (equal). **Hironori Ochi:** Data curation (equal). **Satoshi Yasuda:** Data curation (equal). **Hidenori Toyoda:** Data curation (equal). **Chikara Ogawa:** Data curation (equal). **Takashi Nishimura:** Data curation (equal). **Noritomo Shimada:** Data curation (equal). **Kazuhito Kawata:** Data curation (equal). **Hisashi Kosaka:** Data curation (equal). **Kakizaki Satoru:** Conceptualization (equal); data curation (equal); writing – review and editing (equal). **Takaaki Tanaka:** Data curation (equal). **Hideko Ohama:** Data curation (equal). **Kazuhiro Nouso:** Data curation (equal). **Asahiro Morishita:** Data curation (equal). **Akemi Tsutsui:** Data curation (equal). **Takuya Nagano:** Data curation (equal). **Norio Itokawa:** Data curation (equal). **Tomomi Okubo:** Data curation (equal). **Taeang Arai:** Data curation (equal). **Michitaka Imai:** Data curation (equal). **Yohei Koizumi:** Data curation (equal). **Shinichiro Nakamura:** Data curation (equal). **Masaki Kaibori:** Data curation (equal). **Hiroko Iijima:** Data curation (equal). **Yoichi Hiasa:** Data curation (equal). **Takashi Kumada:** Conceptualization (equal); writing – review and editing (equal).

## CONFLICT OF INTEREST

Takeshi Hatanaka reported honoraria from Eisai. Atsushi Hiraoka reported honoraria from Eli Lilly, Bayer, and Chugai. Toshifumi Tada reported honoraria from AbbVie and Eisai. Satoru Kakizaki is supported by research grants from Abbvie. Takashi Kumada reported honoraria from Eisai. The other authors have nothing to declare.

## STATEMENT OF ETHICS

The studies involving human participants were conducted according to the Declaration of Helsinki. Ethical approval was granted by the institutional review board of Ehime Prefectural Central Hospital (IRB No. 30‐66) (UMIN000043219). Written informed consent for the anonymous use of clinical data was obtained from all patients.

## Supporting information


Figure S1

Figure S2

Figure S3

Figure S4

Figure S5

## Data Availability

The original data underlying the present study may be shared from the corresponding author upon reasonable request.
