# Supplementary figures and images for: The hepatocellular carcinoma modified Gustave Roussy Immune score (HCC‐GRIm score) as a novel prognostic score for patients treated with atezolizumab and bevacizumab: A multicenter retrospective analysis
Source: Cancer Med. 2022 Sep 26;12(4):4259–69. doi: 10.1002/cam4.5294 (PMC9972107; doi:10.1002/cam4.5294)

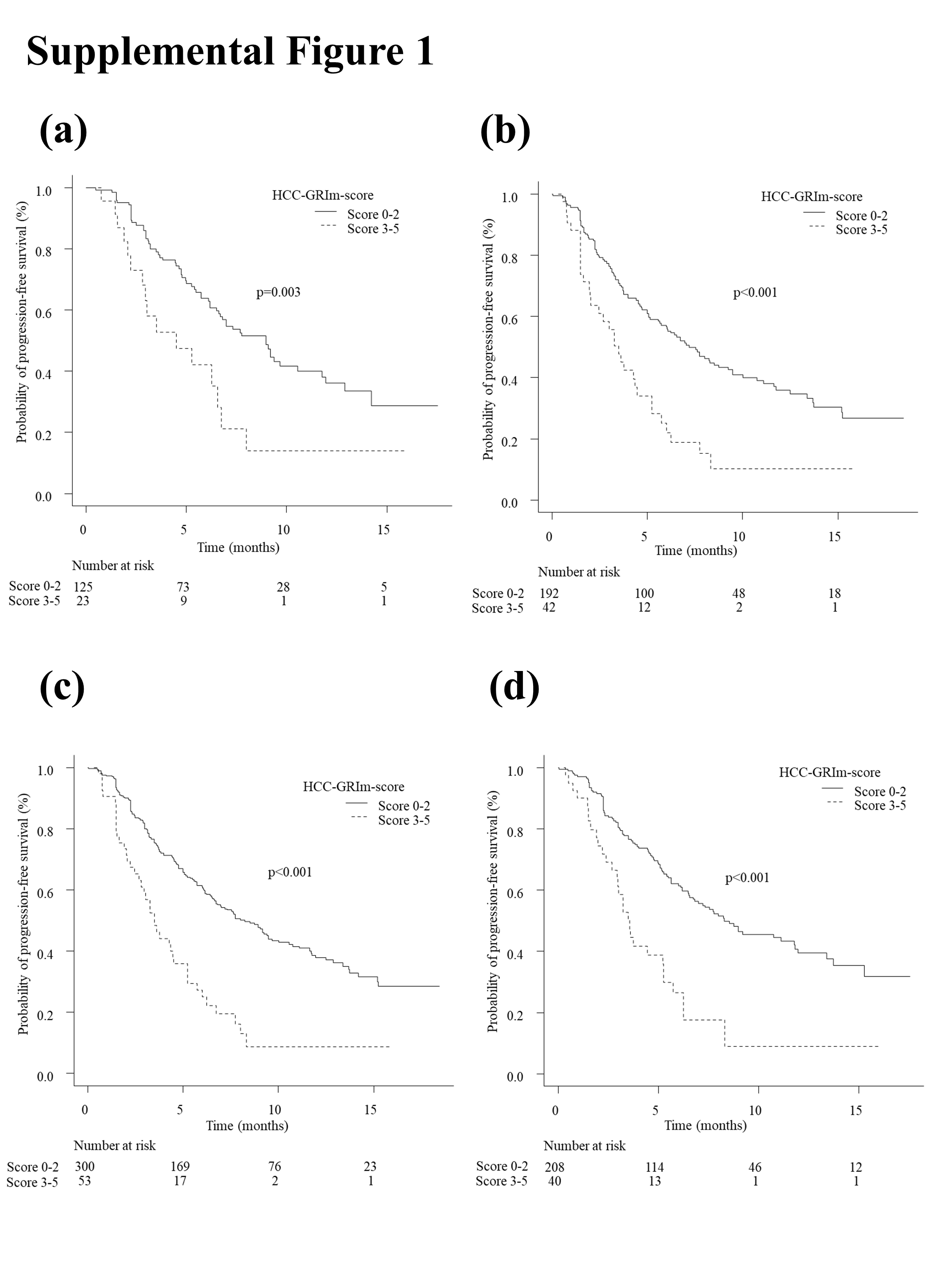

Supplement: Supplementary file 1 — Figure S1 [file CAM4-12-4259-s003.TIF]

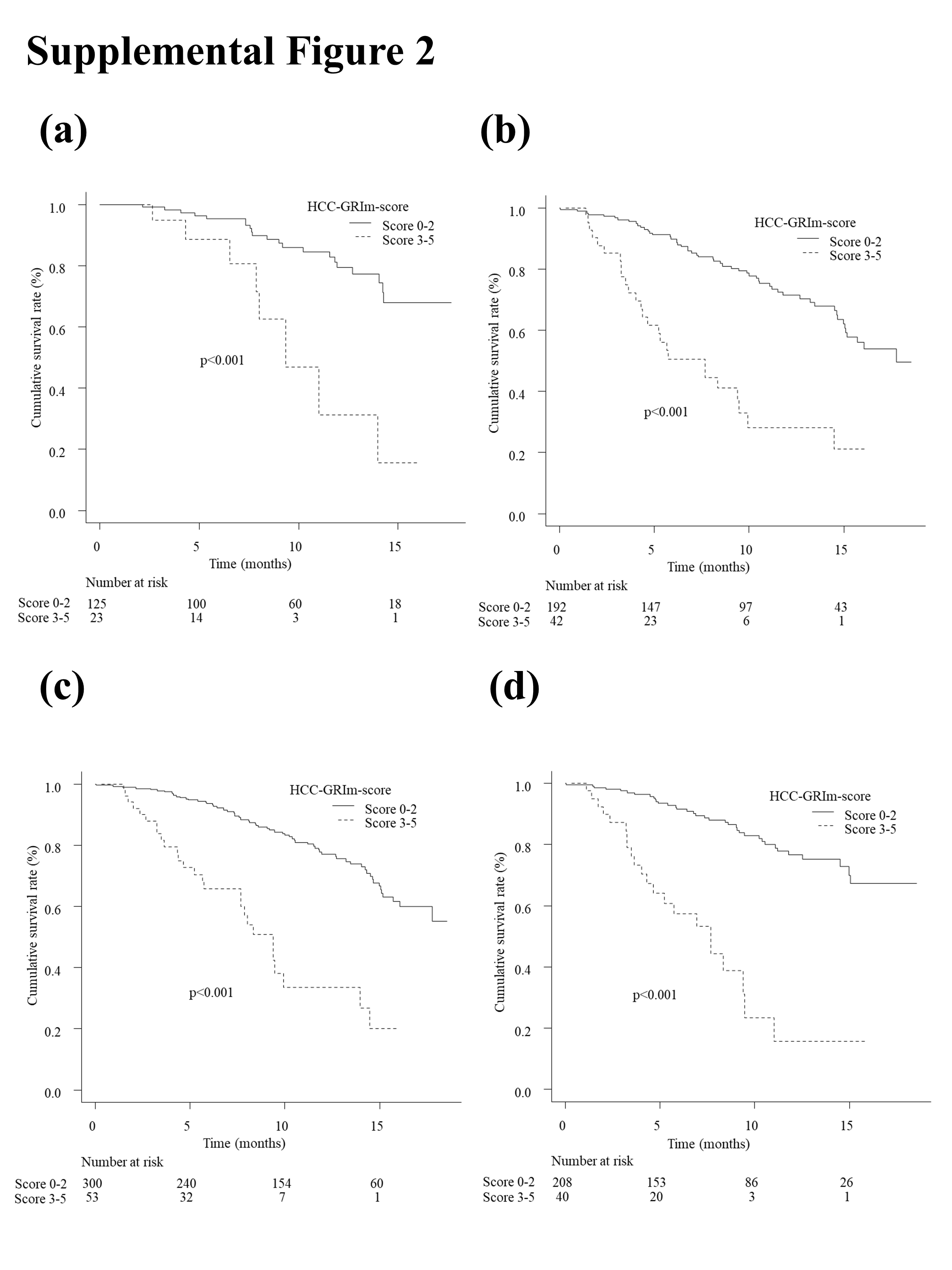

Supplement: Supplementary file 2 — Figure S2 [file CAM4-12-4259-s004.TIF]

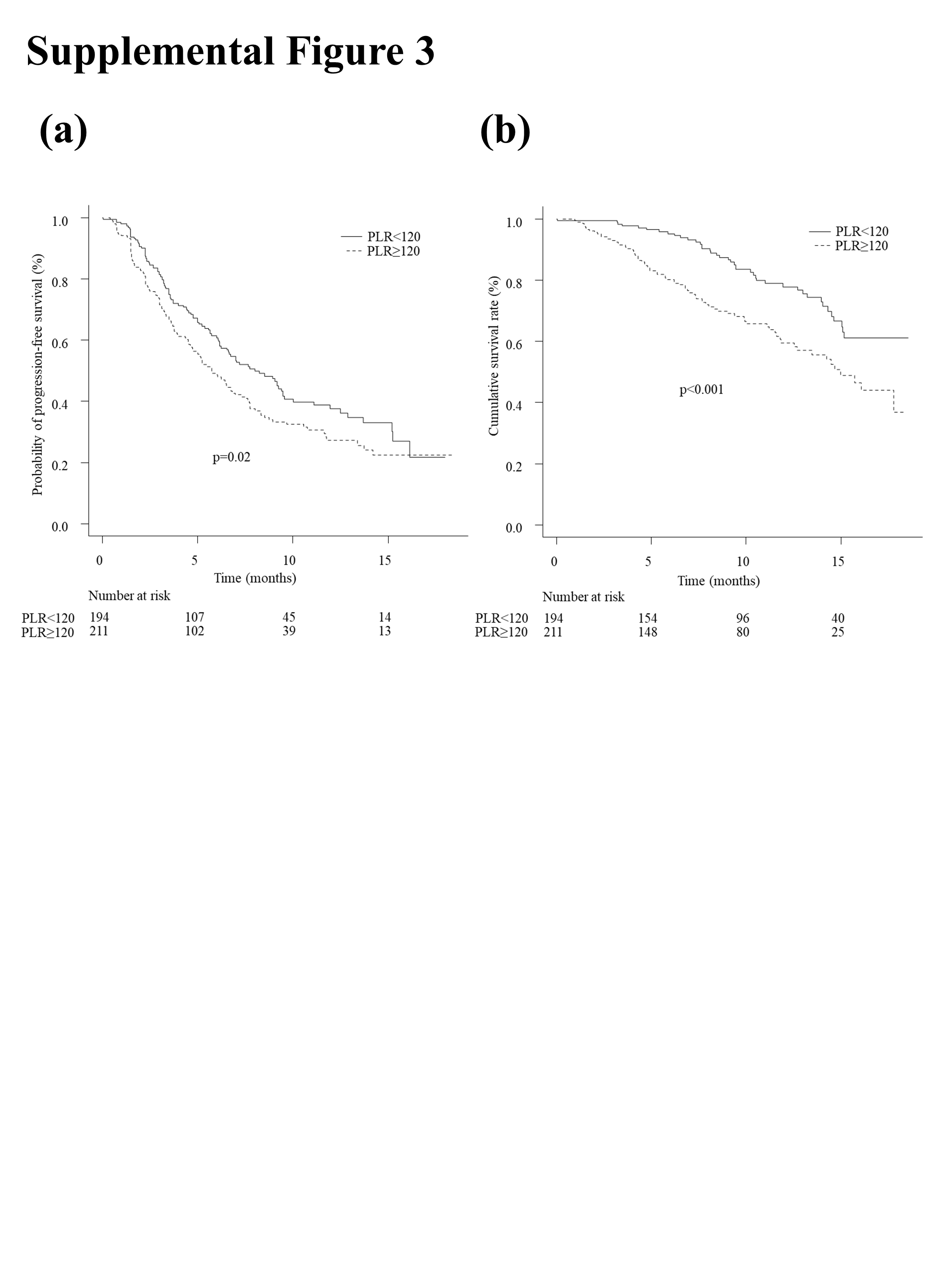

Supplement: Supplementary file 3 — Figure S3 [file CAM4-12-4259-s002.TIF]

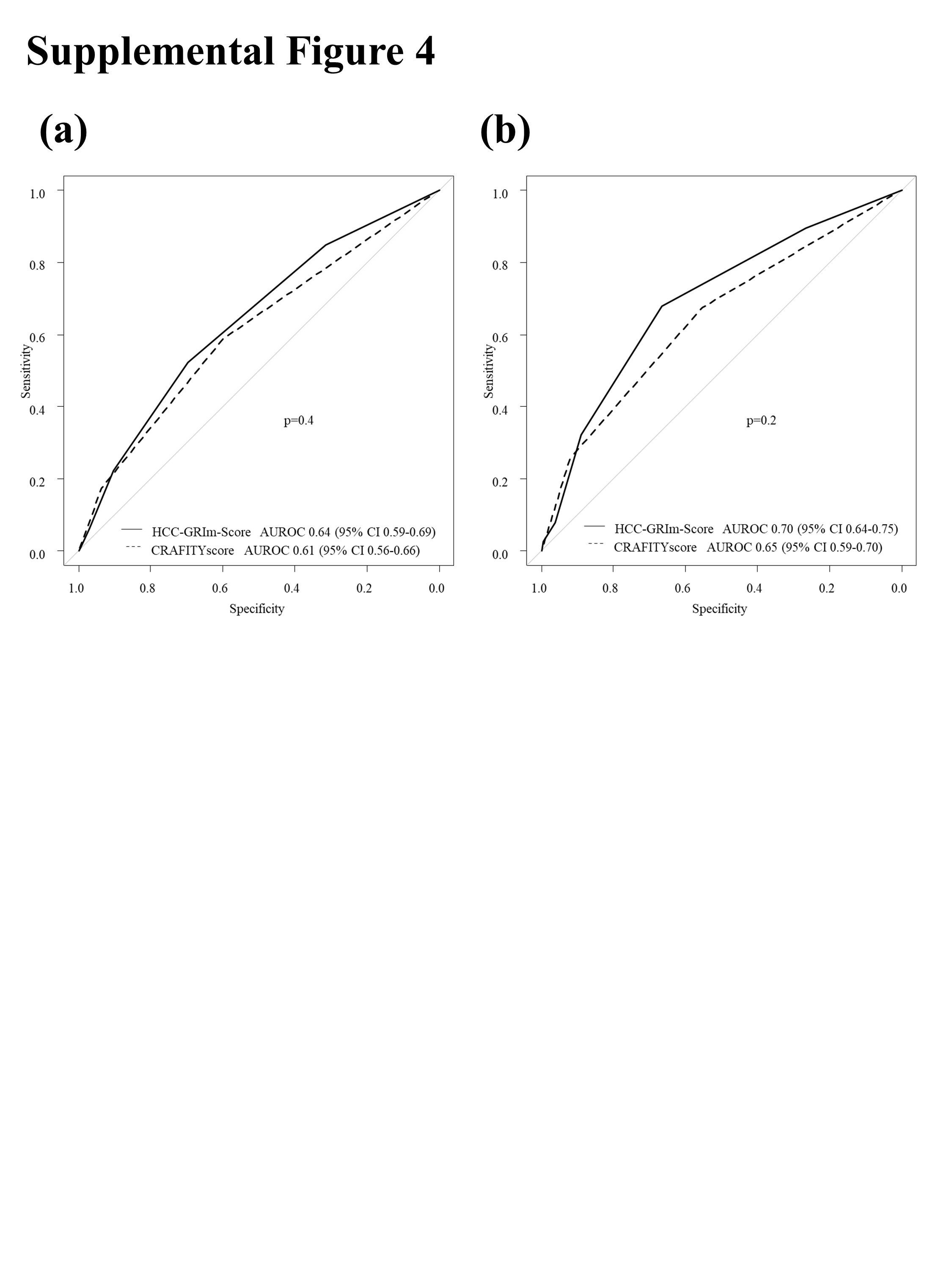

Supplement: Supplementary file 4 — Figure S4 [file CAM4-12-4259-s005.TIF]

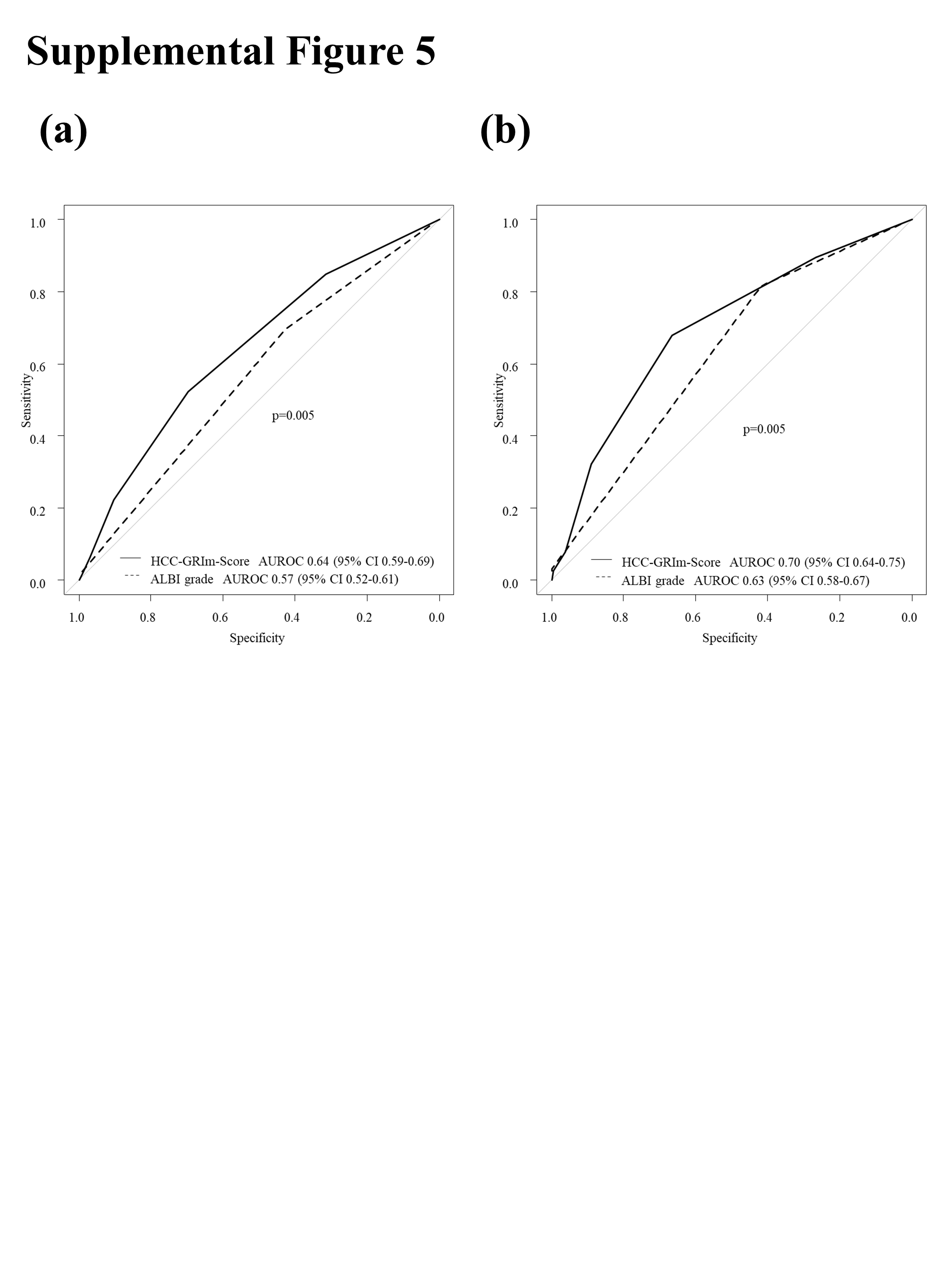

Supplement: Supplementary file 5 — Figure S5 [file CAM4-12-4259-s001.TIF]
